# Metagenomic next-generation sequencing assists in the diagnosis of *Cryptococcus* pneumonia: Case series and literature review

**DOI:** 10.3389/fpubh.2022.971511

**Published:** 2022-11-04

**Authors:** Huifen Wang, Su Yan, Ying Liu, Yaoguang Li, Guangying Cui, Xiaoxu Ma

**Affiliations:** ^1^Department of Infectious Diseases, The First Affiliated Hospital of Zhengzhou University, Zhengzhou, China; ^2^Gene Hospital of Henan Province, The First Affiliated Hospital of Zhengzhou University, Zhengzhou, China; ^3^Precision Medicine Center, The First Affiliated Hospital of Zhengzhou University, Zhengzhou, China; ^4^Health Management Center, The First Affiliated Hospital of Zhengzhou University, Zhengzhou, China; ^5^Department of Respiratory Diseases, The First Affiliated Hospital of Zhengzhou University, Zhengzhou, China

**Keywords:** *Cryptococcus* pneumonia, *Cryptococcus neoformans*, metagenomic next-generation sequencing (mNGS), pathological biopsy, diagnosis

## Abstract

**Background:**

Pulmonary cryptococcosis (PC) was once thought to occur only in patients with immune deficiencies, such as tested positive for the Human Immunodeficiency Virus (HIV). However, in recent years, it has been discovered that more than half of the patients with PC in our nation are individuals with normal immune function. As more and more PC cases are recorded, our diagnosis and treatment approaches, as well as our understanding of PC, are gradually improving. In reality, most PC patients still have a high incidence of misdiagnosis on their initial visit. It is primarily linked to the diverse clinical manifestations, atypical imaging findings, and inaccurate diagnostic approaches.

**Methods:**

The research was conducted from 2019 to 2020. We performed traditional microbiological testing and mNGS on sample from patients with fever of Pulmonary nodules or lung infections. Furthermore, we collected patients' baseline information, clinical features, laboratory and imaging examination results, diagnosis, treatment and outcome. In the end, we confirmed three cases of PC using biopsy and mNGS.

**Conclusion:**

Our data demonstrates that mNGS can be utilized as an auxiliary method for PC diagnosis. Early mNGS aids in the identification of pathogens, enabling early diagnosis and treatment, as well as a reduction in the rate of misdiagnosis and illness progression.

## Introduction

Cryptococcosis is a global mycosis caused by Cryptococcal infection, as well as a prevalent opportunistic infection (1). The common site of infection is the central nervous system (2–4). Pulmonary cryptococcosis (PC) is an acute, subacute, or chronic pulmonary fungal disease caused by *Cryptococcus neoformans* or *Cryptococcus gattii*. The disease is more common in immunosuppressive hosts, particularly those with human immunodeficiency virus (HIV). However, it can also occur to healthy individuals without underlying medical conditions. A study of non-AIDS individuals in China who were pathologically diagnosed with PC revealed that 60% of PC cases were diagnosed in immunocompetent non-HIV patients (5). The prevalence of PC has increased steadily during the past few years. Due to the lack of distinguishable clinical and radiological symptoms, it is simple to misdiagnose a patient or postpone diagnosis. It can often be challenging to identify it from lung tumors, pulmonary tuberculosis, and bacterial pneumonia (4).

The diagnosis of PC mainly depends on histopathological examination and/or specimen etiological smear and culture, such as positive fungal smear or fungal culture in sputum, pharyngeal swab, or bronchoalveolar lavage fluid (BALF) (6–8). If *Cryptococcus neoformans* is detected in blood or puncture cultures, or if the serum cryptococcal capsular polysaccharide antigen latex agglutination test is positive, etiological inquiry can be used to make a clinical diagnosis (9, 10). In general, serum cryptococcal capsular polysaccharide antigen (CrAg) determination can be used to screen for cryptococcosis. This test is routinely repeated in the clinic since it could yield false-negative results in the early stages of cryptococcosis infection. Additionally, patients with mild to moderate pulmonary cryptococcosis who had adequate immune function and little lung lesions had a low positive serum CrAg rate (11). As a result, a novel diagnosis approach is required to aid the diagnosis process.

With the rapid development of sequencing technology and bioinformatics, metagenomic next-generation sequencing (mNGS) has emerged as a new star in clinical diagnosis (12–14). mNGS technology can do high-throughput nucleic acid sequencing in clinical samples without the need for conventional microbe culture, which can then be compared to a database. There is no need for particular amplification since the types of pathogenic microorganisms contained in the samples can be judged based on the comparative sequencing information, and more harmful bacteria in clinical samples can be recognized promptly and reliably. Judging the types of pathogenic microorganisms contained in the samples according to the compared sequence information, there is no need for specific amplification, and more pathogenic microorganisms in clinical samples can be detected quickly and objectively. It is extremely useful in the diagnosis of acute and critical infection (15). In this study, mNGS was used to detect the lung puncture tissues of three patients with probable PC, demonstrating its important role in clinical diagnosis and treatment.

## Materials and methods

Samples were collected from patients according to the standard clinical procedure. DNA is extracted from tissue and quantified. DNA libraries were prepared by using the TruePrep DNA Library Prep Kit V2 for Illumina (Vazyme, Nanjing, China) according to the manufacturer's manuals. The Agilent 2,100 Bioanalyzer (Agilent Technologies, Santa Clara, USA) was used for library quality control. All libraries were pooled with other libraries by using different index sequences and sequenced on an Illumina NextSeq 550Dx platform with the single-end 75bp sequencing option.

Fastq-format data were generated for each sample by using bcl2fastq software (v2.20.0.422, parameters used: –barcode-mismatches 0 –minimum-trimmed-read-length 50). Adapt sequences and low-quality reads were removed using cutadapt v2.10 (-q 25, 25 -m 50). The remaining high-quality reads were first depleted for human sequences by mapping to the human genome (hg38, https://hgdownload.soe.ucsc.edu/downloads.html#human) using bwa-mem 2 v2.1 with default parameters, all unmapped reads were then aligned to the NCBI nt database (https://ftp.ncbi.nlm.nih.gov/genomes/) by using BLAST v2.9.0+ (-task megablast -num_alignments 10 -max_hsps 1 -evalue 1e-10). Alignments were required to be full-length with an identity of at least 95%. A customized Python script was used to identify species-specific alignments. Only the alignments that fulfill the above-mentioned criteria were used for further pathogen identification.

## Results

In this study, all three patients had abnormal lung signs that were indistinguishable from lung cancer and some lung infections. After biopsy and mNGS, we confirmed 3 cases of pulmonary cryptococcus infection. None of these patients presented clinically atypical and there was no evidence of suspected cryptococcus infection. The patient's detailed medical history is described as follows (Tables 1, 2).

**Table 1:** Clinical characteristics of patients.

**Patients NO./Sex/Age/year**	**Final diagnosis**	**Clinical presentation**	**Underlying disease**	**GC**	**ISA**
NO.1/male/42	PC	Back discomfort, dry cough, itchy pharynx dyspnea	None	No	No
NO.2/male/41	PC	No respiratory symptoms	None	No	No
NO.3/male/32	PC	No respiratory symptoms	None	No	No

GC, glucocorticoids; ISA, immunosuppressant.

**Table 2:** Summary of Pathological biopsy results and mNGS results.

**Patient NO**.	**Sample**	**Disease duration of time of diagnostic, day**	**Pathological biopsy testing results**	**mNGS testing results**	**mNGS sequence number**	**Relative abundance**
1	Lung tissue	8	*Cryptococcus neoformans*	*Cryptococcus neoformans*	12,277	99.3%
2	Lung tissue	5	*Cryptococcus neoformans*	No microorganisms were detected		
3	Lung tissue	4	*Cryptococcus neoformans*	*Cryptococcus neoformans*	2,643	

**Figure 1:**
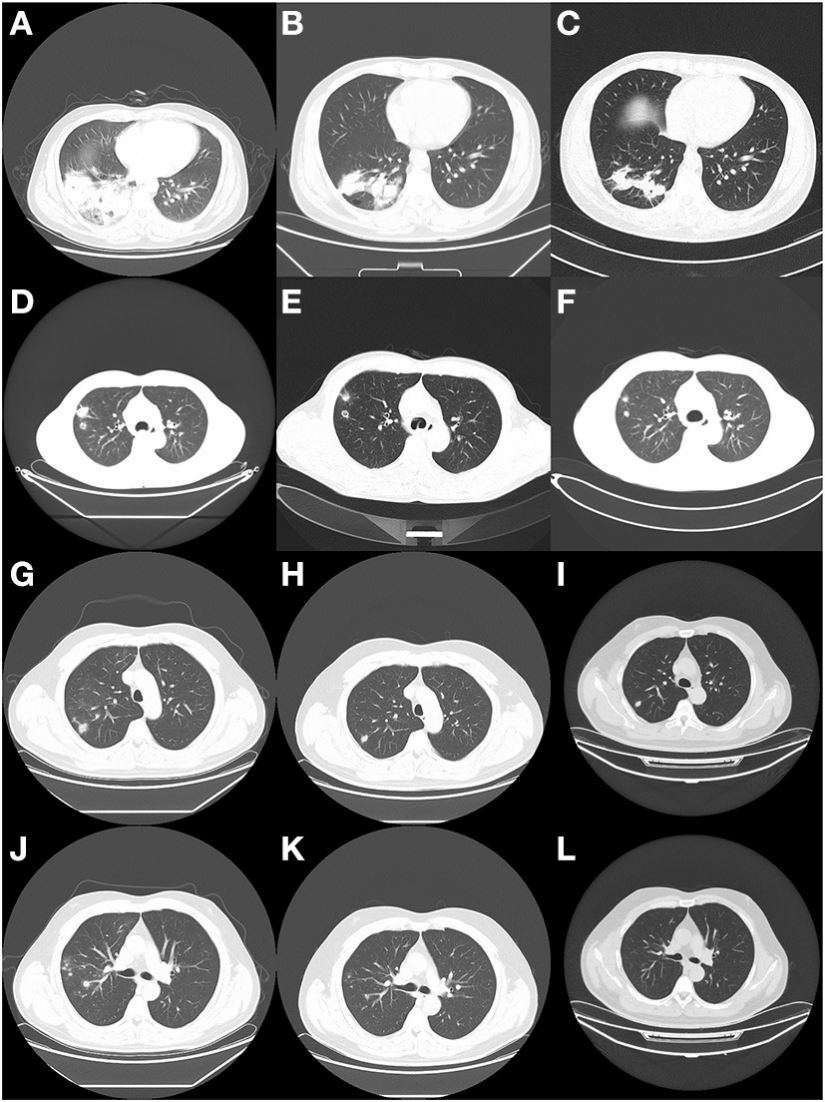
CT findings of the three patients at different periods. **(A)** The enhanced CT scan of patient 1 on the second day of admission showed a right lower lung lesion. **(B)** 1 month after discharge, CT reexamination of patient 1 showed a reduction of the lesion. **(C)** 3 months after discharge, CT reexamination of patient 1 showed a continuous reduction of the lesion. **(D)** CT scan of patient 2 on admission showed lung lesions. **(E)** 2 months after discharge, CT reexamination of patient 2 showed a reduction of the lesion. **(F)** 4 months after discharge, CT reexamination of patient 2 showed a smaller lesion than the previous one. **(G,J)** CT scan of patient 3 at the hospital showed lung lesions. **(H,K)** 6 months later, the patient was reexamined by CT. **(I,L)** 9 months later, the patient was reexamined by CT.

**Figure 2:**
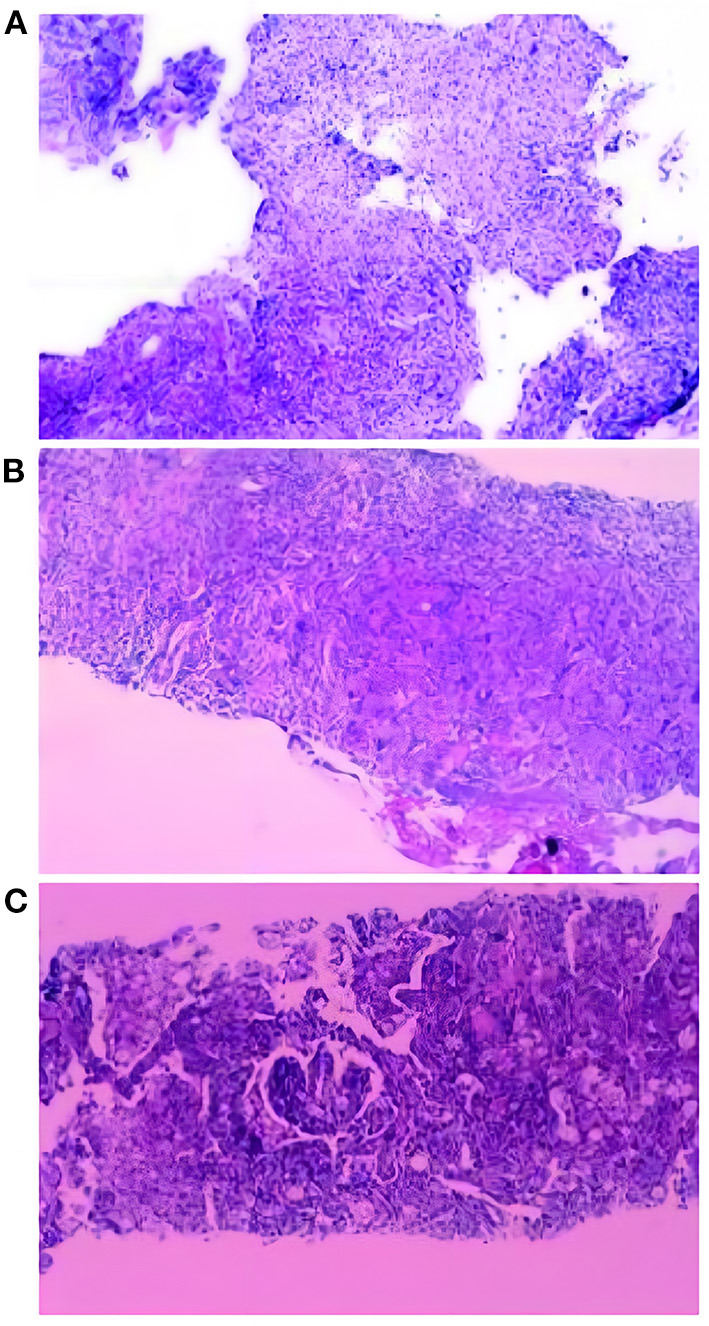
Histopathological examination results of three patients. **(A)** Granulomatous inflammation, with more spore-like material, Manifestations of fungal infection, tends to cryptococcus neoformans. Special staining: PAS (+), AFB (-), GMS (+). **(B)** Lung tissue chronic inflammation, local multinucleated giant cells, intracellular spore-like structure, Special staining: PAS (+), AFB (-), GMS (+). Combining the results of the special stain and the clinical features of the patient, the patient was considered to have a cryptococcal infection of the lung. **(C)** Combined with the morphology and special staining results, Tendency to fungal infections: *Cryptococcus*and *Talaromyces marneffei* infection. Pneumocystis *Pneumocystis jirovecii*infection cannot be ruled out. Special dyeing: Mucus red card (+), GMS (+), PAS (+).

Patient 1 is a 42-year-old male, farmer in Xuchang City, Henan Province. He was admitted to our hospital (the First Affiliated Hospital of Zhengzhou University) with “back discomfort for 3 months and cough for more than 1 month” as the chief complaint On March 17, 2020. The specific clinical manifestations are chronic pain in the right back, accompanied by itchy throat and dry cough, without other uncomfortable symptoms. CT of the other hospital revealed a high-density shadow and partial consolidation in the lower lobe of the right lung, as well as patches of infected air-containing lung tissue. CRP (C-reaction protein) and white blood corpuscles were also high. Because the efficacy of the empirical anti-infective treatment was unsatisfactory, he was transferred to our hospital. Physical examination reveals increased breathing movement, decreased breathing sound in the right lower lung, enhanced voice resonance, rough breathing sound in the right lung, and a fast heart rate of around 110 beats per min that is uniform. Laboratory test findings that are abnormal: Blood Coagulation: Fib 6.12 g/L, D-Dimer 0.85 mg/L; Glucose 6.32 mmol/L, GGT 87U/L, ALP 160U/L, ADA 23U/L, TG 1.85 mmol/L; Urinalysis: GLU ++, Unclassified crystal 40; CBC: WBC 12.6 × 10^∧^9/L, Hb129.0 g/L; Neut% 85.6%, Lymph% 7.2%; Cell compartment activation: T lymph 637/μ, T (CD4+) 339/μL; CRP:15.90mg/L. CT (Computed Tomography) of the chest (2020/03/18) (Figure 1A): Lesion in the right lower lobe, considering inflammation and a lesion in the right lower lobe. Bronchoscopy: There were a few secretions in the airway, but the etiological examination of lavage fluid revealed nothing unusual. Anti-infective treatment was given with piperacillin Tazobactam was attempted, but the results were disappointing. Bronchoalveolar lavage fluid was used for bacterial smear staining and culture, and the results were negative. The patient was advised to use BALF (Bronchoalveolar Lavage Fluid) for mNGS testing, but the patient's family refused. On the 6th day after admission, the patient had a CT-guided lung biopsy, and the punctured tissue was examined for pathology (Figure 2A) and mNGS simultaneously (Illumina Next550, San Diego, USA). *Cryptococcus neoformans* was detected by pathological examination and mNGS. The result of mNGS was available 2 days before the pathological examination. mNGS results showed that the sequence number of *Cryptococcus* was 12,489 and the relative abundance was 99.3%. The patient was diagnosed with PC based on histology, mNGS, and later identification of cryptococcal capsular polysaccharide antigen. Instead, voriconazole was used as an antifungal medication. The patient was discharged after remission of symptoms. The CT of outpatient reexamination was satisfactory 1 and 3 months after discharge (Figures 1B,C).

Patient 2 is a 41-year-old male farmer from Luohe City, Henan Province. The patient was taken to the hospital on October 8, 2020, due to a “physical examination found right lung nodule for 10 days.” During the hemorrhoidectomy in the local hospital more than 10 days ago, the patient was found to have right lung nodules by chest CT. However, he did not have any uncomfortable clinical symptoms. Check result that is abnormal: CBC: thrombocytocrit 0.29%; Blood Coagulation: D-Dimer1.73 mg/L, Urinalysis: Occult blood++, protein+, RBC17/μL, WBC 88/μL, Unclassified crystal 71/μL; Liver function: TP 55.3. G test, CRP, PCT, and tumor markers were all normal. Judging from the basic data, the possibility of lung cancer cannot be ruled out. On the second day after admission, the patient had a CT-guided lung puncture biopsy and mNGS detection (Figure 1D). mNGS showed that no microorganism was detected. PATHO: Initial report (2020/10/12) Chronic inflammation of the lung tissue can be detected, as well as spindle cell growth and focal necrosis, as well as multinucleated giant cells. Second Report (2020/10/13) Lung tissue chronic inflammation, local multinucleated giant cells, intracellular spore-like structure, Cryptococcal infection is considered. Special staining: PAS (+), AFB (-), GMS (+). Cryptococcal capsular polysaccharide antigen (GXM): 71.949(+). C*ryptococcus neoformans* pneumonia was suspected based on pathological findings and the GXM test, and fluconazole was administered orally. The cerebrospinal fluid was sent for examination following lumbar puncture to evaluate whether there was a cryptococcal infection in the brain, and the results showed that Ink stain (-), AFB (-) (Figure 2B). Cerebrospinal fluid cytology revealed an increase in monocytes, indicating that Cryptococcal meningitis had been ruled out. The patient is generally in good condition and does not express any discomfort. At the request of the patient, he was discharged from the hospital on October 18, 2020, and continued oral fluconazole medication outside of the hospital. 2 and 4 months after discharge, the CT revealed good results (Figures 1E,F).

Patient 3 is a skilled worker 32-year-old middle-aged male from Zhengzhou, Henan Province. The patient was admitted to the hospital on December 23, 2019, because of the “physical examination found right lung nodular shadow for 1 month.” During the physical examination before 1 month, the patient found right lung nodule shadow, no cough, sputum, chest pain, no dyspnea, no hemoptysis; CT (another hospital): multiple nodules of various sizes could be seen in the middle and upper lobe of the right lung, and some of the edges were irregular. CT (our hospital): Multiple tiny nodules and mass shadows in the right upper lung, considering infection (Figures 1G,J). Results of laboratory examination: ABG: PH 7.41, pCO2 41.0 mmHg, pO2 84.0 mmHg, GLU 5.4 mmol/L; CBC: WBC 8.39 × 10^∧^9/L, RBC 5.37 × 10^∧^12/L, Hb 161.8 g/L, PLT 264 × 10^∧^9/L. Urinalysis, Stool Routine, G test, CRP, PCT, T-SPORT, Tuberculous sputum smear, and tumor marker all were negative. A CT-guided lung biopsy was performed on the third day of admission. PATHO: First report (2019/12/27): Prefer cryptococcal infection, to be reported after histochemical staining. Second Report (2020/01/2): Combined with the morphology and special staining results, the preference was *Cryptococcus* with *Talaromyces marneffei* infection. *Pneumocystis jirovecii* infection cannot be ruled out. Special dyeing: Mucus red card (+), GMS (+), PAS (+) (Figure 2C). mNGS (2020/01/11): *Cryptococcus neoformans*. mNGS results showed that the sequence number of *Cryptococcus* was 2,680. After diagnosis, the treatment drug was added to fluconazole and Compound Sulfamethoxazole based on Rifamycin Sodium Injection, moxifloxacin, and the patient was discharged after the symptoms improved. CT scans taken 6 and 9 months later revealed a steady improvement (Figures 1H,I,K,L).

## Discussion and literature review

In 1894, an organism similar to Genus *Saccharomyces* was isolated from a young woman with a bone infection. Not long after, a similar strain was isolated from peach juice and given the name “Saccharomyces Neoformans” due to the unique colony morphology it had. Because the parasite was unable to develop ascospores, a feature of yeast, it was eventually given the name “Cryptococcus neoformans” in 1901 (16, 17). They are primarily discovered in dirt, decaying wood, and pigeon droppings in tropical and subtropical regions. Inhaled *Cryptococcus neoformans* can invade local lung tissue and cause acute, subacute, or chronic lung infection (18, 19). Although it can afflict anyone at any age, young and middle-aged males are the most frequently affected (20, 21). The main symptoms of the disease include fever, cough, expectoration, chest pain, a small number of patients have hemoptysis. Even patients with healthy immune systems lacked respiratory symptoms (22, 23). Therefore, the clinical manifestations of the patients are not specific. Single or numerous nodular or mass shadows, frequently observed under the pleura, as well as burr or halo indications might be recognized as imaging characteristics of the disease. Nodules or cavities in masses are frequently developed in immune-competent hosts, followed by pulmonary parenchyma infiltration that is difficult to distinguish from other forms of bacterial pneumonia (24). According to international studies, the proportion of non-immunodeficient hosts in patients with cryptococcosis patients is often <35% (25, 26). However, compared with foreign countries, non-immunodeficient hosts account for a higher proportion of cryptococcosis patients in China (27). Moreover, there are regional differences in incidence, with the south having a higher rate than the north (28). The three PC patients in our study were all normal middle-aged males from the north, immunologically normal, and without a history of travel. In line with earlier studies, the three patients' CT scans were similarly non-specific, making it challenging to distinguish between lung cancer, pulmonary tuberculosis, and other types of pneumonia. Further to that, because this disease is complex and occult, and it is not a common type of pneumonia, it is easy to overlook in the clinic. Therefore, all three patients in this study were detected Cryptococcus infection unexpectedly.

For the diagnosis of PC, the commonly used clinical etiological diagnosis methods of cryptococcosis include traditional smear examination, isolation and culture, CrAg detection, and histopathological examination. In the majority of cases, the results of a histological investigation are used to determine the diagnosis. The percentage of instances that can be determined by smear and culture results is extremely low, especially for people with normal immunity. The sensitivity of serum CrAg detection is mainly affected by infection site and infection type. Some studies have pointed out that the sensitivity and specificity of serum CrAg detection in cryptococcal meningitis and disseminated cryptococcal infection can reach 93~ 100% and 93~ 98% (4). However, the sensitivity rate in HIV-negative patients with single pulmonary cryptococcosis is only 25~ 56% (8). As a result, serum CrAg screening may result in false negative results. Additionally, it has been discovered that the host's immunological status and serum CrAg sensitivity are connected (29–31).

In 2014, the New England Journal of Medicine published the first clinical case of leptospirosis diagnosed by mNGS, which opened the prelude to the detection of pathogens by mNGS (32). In the years that followed, mNGS made great strides in the discovery of new pathogens, the detection of rare and significant pathogens, and clinical research leveraging big data. One example of this is the use of mNGS to support the clinical diagnosis of liver tuberculosis. The timely use of mNGS helps the clinic to identify the cause of fever accurately and quickly, and it promotes accurate clinical diagnosis (33). In the detection of pathogens, antibiotics have a large influence on the traditional culture, but the detection of mNGS is rarely affected by antibiotics and has high sensitivity (34). When patients with pulmonary infection are admitted to the hospital, they are frequently treated with empirical antibiotics, especially if the infection is severe. And different patients have different host factors, imaging changes, and laboratory results, some patients have long-term use of hormones, which may affect the traditional pathogen identification culture, but mNGS can compensate for these flaws. When patients are hospitalized and given antibiotic treatment, it may prevent some harmful microbes from being detected by bacterial culture. In addition, the time point for bacterial culture is also very strict, which are the disadvantages of traditional culture. At the same time, the number of days spent in the hospital prior to mNGS has no effect on the pathogen detection rate in mNGS, extending the detection range to some extent. In addition, some studies have shown that mNGS is more sensitive than conventional culture in the detection of infectious bacteria, fungi, and unclassified pulmonary pathogens in transbronchial lung biopsy (TBLB), bronchoalveolar lavage fluid (BALF), and bronchial needle brushing (BB) samples, with no difference in sensitivity among the three clinical samples. TBLB had the greatest specificity, followed by BB and BALF (35). All three patients in this study were examined with lung puncture tissue for mNGS, no microorganism was detected in one case. At that time, because the results of pathological biopsy could be diagnosed, no further tests were needed. In addition, the patient declined the second mNGS test because of the good results after changing the therapeutic drugs. As for the reasons for the negative mNGS test, there may be two aspects in our analysis: The thick-walled capsule surrounded by *Cryptococcus neoformans in vitro* is the most prominent morphological feature of it. The three primary components of the Cryptococcus neoformans capsule are mannoprotein, galactoxylomannan, and glucuronoxylomannan (GXM). The phagocytosis and killing effect of macrophages is an important link in the innate immunity mechanism. It has been found that the capsule volume of *Cryptococcus neoformans* is negatively correlated with antibody and complement-mediated phagocytosis of macrophages. That is, the larger the capsule, the less likely it is to be swallowed (36–38). And some studies have shown that *Cryptococcus neoformans* phagocytized by macrophages can greatly increase the capsule volume, as the capsule is enlarged, the density of the original capsule in the inner layer increases, which can significantly reduce the penetration of exogenous substances (39). The enlarged capsule can help it resist the killing effect of nitrogen and oxygen free radicals in macrophages, which is proportional to the size of the capsule. Because it takes longer to digest the thicker capsule following phagocytosis of Cryptococcus neoformans, we thus hypothesized that the capsule may still exist at the time of mNGS while the internal gene has been lost. In addition, due to the thick capsule of *Cryptococcus neoformans*, mNGS detection may be hampered by wall-breaking and nucleic acid extraction failure, resulting in the failure to detect the gene sequence. mNGS results for human samples typically contain 95 percent human readings, with pathogen readings accounting for only a minor portion of all sequencing outcomes (13, 40). Looked in another way, the sample we used was lung biopsy tissue, nd it contained an excessive number of human readings. The high proportion of host DNA reduces the sensitivity of metagenomics sequencing to pathogenic microorganisms, mainly because the high background of human sequences present in large numbers during sequencing will mask the pathogenic microbial signal in low microbiome specimens. And the high level of host DNA results in a large amount of human sequence data being generated when the sample is taken off the machine, which is particularly noticeable for low abundance pathogenic microbial samples. Therefore, tissue samples are generally less positive than BALF samples, which are low in human sources. We reviewed the sample information for case 2 and found that it was sequenced at 48 M, with a high proportion of human reads of over 99.82%, so we suspect that the pathogenic microbial signal in case 2 was masked by the high number of human reads.

As a result, eliminating human DNA sequences from pathogen-rich materials is critical for using mNGS in diagnostic microbiology (13, 18, 40). It can be noted that each of the regularly utilized clinical diagnostic procedures for *Cryptococcus neoformans* has its own set of benefits and drawbacks. CrAg detection is non-invasive and has high sensitivity and specificity, but the disadvantage is that patients with normal host immune function are prone to false negatives. Furthermore, this test is typically only performed by clinicians as a screening procedure when they have a suspicion of the illness. Although the pathological examination is the gold standard of diagnosis, it is also to some extent subjective, as the reporting physician needs to draw conclusions from morphological observations of the specimen. And this is intimately tied to the doctor's individual evaluation, perception and comprehension of the disease. mNGS can quickly detect pathogens and help doctors make judgments. But it cannot be regarded as the gold standard of diagnosis. More research is needed to prove the diagnostic efficacy of mNGS for PC. For some difficult to diagnose PC, especially like the patients in this study, the symptoms are not typical and difficult to distinguish from lung cancer. Furthermore, because there are no suspicious PC factors, it is difficult to detect PC clinically. At this time, mNGS, which can detect a wide spectrum of pathogenic bacteria, may be useful in assisting patients in identifying the pathogen that is causing the infection. In addition, there are several limitations to mNGS. In some less developed areas, such as countries with a high incidence of cryptococcosis but limited resources, the technology is not available to most routine laboratories. However, with the development of this technology in recent years, its cost has gradually decreased, and together with the simple availability of the required samples, mNGS will reach more and more locations (41).

## Conclusion

To summarize, while many diagnostic approaches for the diagnosis of *Cryptococcus neoformans* are not ideal, they are complementary. mNGS can be utilized as a supplementary diagnosis of *Cryptococcus neoformans* as a new diagnostic approach. As mNGS becomes more affordable (42–44), we anticipate it will be used in conjunction with traditional diagnostic methods to diagnose PC more rapidly and precisely, as well as to aid the selection of most appropriate antifungal medications.

## Data availability statement

The datasets for this article are not publicly available due to concerns regarding participant/patient anonymity. Requests to access the datasets should be directed to the corresponding authors.

## Ethics statement

Written informed consent was obtained from the individual(s) for the publication of any potentially identifiable images or data included in this article.

## Author contributions

XM and GC designed the study. HW, YLiu, and SY collected clinical data. HW and YLi analyzed the data. HW, XM, and GC wrote the manuscript. All authors reviewed and approved the manuscript.

## Funding

This research was equally funded and supported by the China Postdoctoral Science Foundation (2020T130109ZX), Henan Province Science and Technology Project (GC).

## Conflict of interest

The authors declare that the research was conducted in the absence of any commercial or financial relationships that could be construed as a potential conflict of interest.

## Publisher's note

All claims expressed in this article are solely those of the authors and do not necessarily represent those of their affiliated organizations, or those of the publisher, the editors and the reviewers. Any product that may be evaluated in this article, or claim that may be made by its manufacturer, is not guaranteed or endorsed by the publisher.
